# Agonist mediated internalization of M_2 _mAChR is β-arrestin-dependent

**DOI:** 10.1186/1750-2187-1-7

**Published:** 2006-12-05

**Authors:** Kymry T Jones, Maria Echeverry, Valerie A Mosser, Alicia Gates, Darrell A Jackson

**Affiliations:** 1 School of Biology, Georgia Institute of Technology, Atlanta, GA 30332 USA; 2 Laboratorio de Parasitologia (301), Facultad de Medicina, Universidad Nacional de Colombia, Bogotá, Columbia; 3 Department of Anatomy and Neurobiology, Neuroscience Institute, Morehouse School of Medicine, Atlanta, GA 30310 USA; 4 Department of Biomedical and Pharmaceutical Sciences, University of Montana, Missoula, MT 59812 USA

## Abstract

**Background:**

Muscarinic acetylcholine receptors (mAChRs) undergo agonist-promoted internalization, but evidence suggesting that the mechanism of internalization is β-arrestin dependent has been contradictory and unclear. Previous studies using heterologous over-expression of wild type or dominant-negative forms of β-arrestins have reported that agonist-promoted internalization of M_2 _mAChRs is a β-arrestin- and clathrin-independent phenomenon. In order to circumvent the complications associated with the presence of endogenous β-arrestin that may have existed in these earlier studies, we examined agonist-promoted internalization of the M_2 _mAChR in mouse embryonic fibroblasts (MEFs) derived from β-arrestin knockout mice that lack expression of either one or both isoforms of β-arrestin (β-arrestin 1 and 2).

**Results:**

In wild type MEF cells transiently expressing M_2 _mAChRs, 40% of surface M_2 _mAChRs underwent internalization and sorted into intracellular compartments following agonist stimulation. In contrast, M_2 _mAChRs failed to undergo internalization and sorting into intracellular compartments in MEF β-arrestin double knockout cells following agonist stimulation. In double knockout cells, expression of either β-arrestin 1 or 2 isoforms resulted in rescue of agonist-promoted internalization. Stimulation of M_2 _mAChRs led to a stable co-localization with GFP-tagged β-arrestin within endocytic structures in multiple cell lines; the compartment to which β-arrestin localized was determined to be the early endosome. Agonist-promoted internalization of M_2 _mAChRs was moderately rescued in MEF β-arrestin 1 and 2 double knockout cells expressing exogenous arrestin mutants that were selectively defective in interactions with clathrin (β-arrestin 2 ΔLIELD), AP-2 (β-arrestin 2-F391A), or both clathrin/AP-2. Expression of a truncated carboxy-terminal region of β-arrestin 1 (319–418) completely abrogated agonist-promoted internalization of M_2 _mAChRs in wild type MEF cells.

**Conclusion:**

In summary, this study demonstrates that agonist-promoted internalization of M_2 _mAChRs is β-arrestin- and clathrin-dependent, and that the receptor stably co-localizes with β-arrestin in early endosomal vesicles.

## Background

Muscarinic acetylcholine receptors belong to the superfamily of G-protein coupled receptors (GPCRs) that are commonly expressed in a variety of tissues and are classified into five known subtypes (M_1 _-M_5 _mAChR). M_1_, M_3_, and M_5 _mAChRs are selectively coupled to G_q _proteins while M_2 _and M_4 _mAChRs are linked to G_i_/G_0 _proteins [1,2]. M_2 _mAChRs are the primary muscarinic subtype in the heart where their stimulation leads to the regulation of myocardial contractility [3]. As with other GPCRs, M_2 _mAChR activity is tightly regulated by desensitization and internalization. These regulatory mechanisms are typically associated with receptor phosphorylation followed by either recycling or down-regulation [4-9].

Desensitization is a complex process that involves agonist-dependent phosphorylation at specific serine/threonine residues by G-protein-coupled receptor kinases (GRKs) followed by β-arrestin binding. Two widely expressed isoforms of β-arrestin (1 and 2) are known to be involved in uncoupling receptors from their cognate G-proteins thereby attenuating receptor signalling [10,11]. Typically, agonist-induced phosphorylation facilitates receptor internalization, which serves to either resensitize or down-regulate desensitized receptors [12]. β-arrestins have been shown to facilitate internalization by directly interacting with the β_2 _subunit of the clathrin-AP2 (adaptor protein 2) complex and clathrin itself [11,13]. Thus, β-arrestins can induce receptor sequestration by directly interacting with the endocytic machinery. Many receptors such as the prototypic β_2_-adrenergic receptor (β_2_AR) internalize in a clathrin and β-arrestin dependent fashion. Hence, β-arrestin facilitates clathrin-mediated endocytosis [11,13].

In addition to desensitization and internalization, β-arrestins are known to play a role in other cellular processes that include intracellular trafficking and signalling [12]. Association of β-arrestin with agonist-occupied receptors has been shown to initiate intracellular signalling by functioning as an assembly site for signalling components such as Src, JNK3, and ERK1/2 [14-17]. Therefore, β-arrestin-receptor complexes can lead to cytosolic retention and activation of signalling molecules following receptor-mediated signalling at the cell surface. The physiological roles of this process include decreasing cell proliferation and regulating cytoskeletal rearrangements by spatially restricting ERK activation to the cytosol [16,18]. Recent reports have also suggested that β-arrestins can function at post-endocytic stages to regulate receptor sorting. It has been shown that receptors exhibit differential affinities for β-arrestin and therefore are classified into two groups [19]. Class A receptors (including β_2_AR and dopamine receptors) are thought to interact with β-arrestin at the plasma membrane but immediately disassociate following localization to clathrin-coated pits. Hence receptors enter early endosomes devoid of β-arrestin and are typically resensitized and rapidly recycled [20]. In contrast, Class B receptors (vasopressin-V_2_R, angiotensin-AT_1A_R, and neurotensin receptors) stably associate with β-arrestin so that β-arrestin/receptor complexes remain intact and are internalized into juxtanuclear endosomal compartments [21]. This interaction can persist for prolonged periods of time. This stable association may dictate the kinetics of receptor recycling since AT_1A_R and V_2_R recycle very slowly [20,21]. A functional consequence of β-arrestin association may also be to facilitate receptor down-regulation.

The role of β-arrestins in regulating the trafficking of M_2 _mAChRs has been contradictory and unclear. Reports have demonstrated that phosphorylation by GRK2 on serine/threonine residues in the third intracellular loop of M_2 _mAChRs recruits β-arrestin and leads to receptor desensitization and subsequent internalization [7].

Whether β-arrestin is involved directly in agonist-promoted endocytosis of M_2 _mAChRs remains unclear. Indeed over-expression of β-arrestin has been reported to increase agonist-promoted internalization of M_2 _mAChRs but not of M_1 _or M_3 _mAChRs [22]. Furthermore, Claing *et al*. have shown that M_2 _mAChRs internalize in a dynamin- and β-arrestin-insensitive manner when expressed in HEK293 cells [23]. Others have reported that the Arf6 GTPase (ADP-ribosylation factor 6) facilitates M_2 _mAChR entry into primary vesicles, which fuse with clathrin-derived early endosomes [24,25]. These data do not necessarily rule out β-arrestin as a regulator in agonist-promoted endocytosis of M_2 _mAChRs. Therefore, to clarify whether agonist-promoted internalization of M_2 _mAChRs is arrestin dependent, we utilized mouse embryonic fibroblasts (MEFs) derived from β-arrestin null mice that lack expression of one or both isoforms (β-arrestin 1 and 2) and their wild type littermates as control cells [26]. Here we report that agonist-promoted internalization of M_2 _mAChRs is β-arrestin dependent and M_2 _mAChRs form stable complexes with β-arrestin at the early endosome. Furthermore, we demonstrate that agonist-promoted internalization of M_2 _mAChRs is clathrin-dependent. These results suggest that β-arrestin plays an important role in regulating M_2 _mAChR activity.

## Results

**Figure 1:**
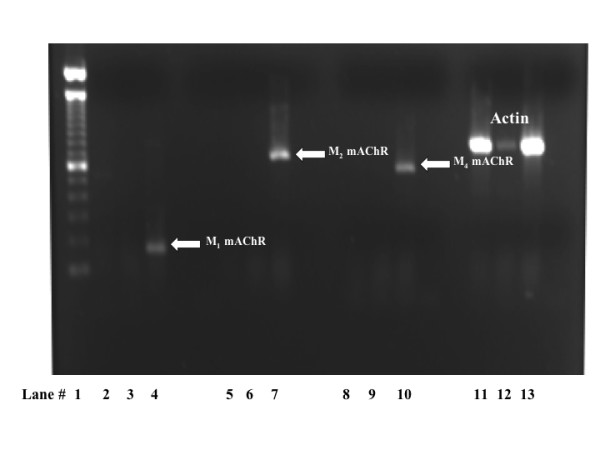
**Mouse embryonic fibroblasts (MEF) cells do not express mRNA encoding M_1_, M_2 _or M_4 _mAChR subtypes**. A representative gel showing lack of mAChR expression in MEF wild type and KO1/2 cells Lanes consisted of 100 bp ladder (lane 1), wild type MEF cells (lanes 2, 5, 8 and 11), MEF β-arrestin KO1/2 (lanes 3, 6, 9, and 12), rat cortex (lanes 4 and 10 and 13), and rat cerebellum (lane 7). The white arrows point to M_1_, M_2 _and M_4 _mAChR PCR product from cDNA as positive controls. The electrophoresis gel shown is a representative of at least 3 independent experiments.

To determine whether the MEF cells used in this study expressed endogenous mAChRs, we performed RT-PCR aimed at detecting mRNA encoding M_1_, M_2 _and M_4 _mAChR subtypes. As positive controls, we used postnatal rat cerebellum tissue for M_2 _mAChR mRNA and postnatal rat cortical tissue for M_1 _and M_4 _mAChR mRNA. RT-PCR analysis clearly demonstrated that MEF wild type as well as MEF double knockout cells (MEF KO1/2) did not express mRNA encoding M_1_, M_2_, or M_4 _mAChR subtypes (Fig. 1). Accordingly, radioligand-binding assays also confirmed that MEF wild type as well as MEF KO1/2 did not express mAChRs at any detectable level (*data not shown*). Therefore, we concluded that MEF cells do not express endogenous mAChRs.

**Figure 2:**
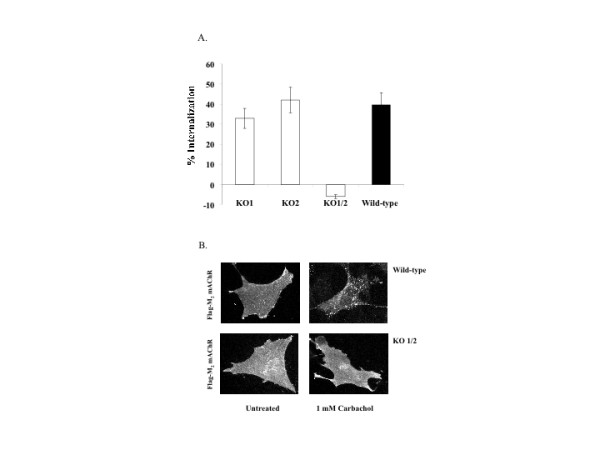
**Agonist-promoted internalization of M_2 _mAChR in MEFs is β-arrestin-dependent**. A.) Approximately 24 h following transfection with M_2 _mAChR, cells were stimulated with 1 mM carbachol for 1 h and agonist-promoted internalization was determined using [^3^H-NMS]. Data are presented as the mean ± standard error from 3 separate experiments with each experiment consisting of 8 to 11 independent determinations. Statistical test was performed using ANOVA with the post hoc Bonferroni/Dunn test (asterisk indicates * p < 0.001). B.) Cells were transfected as described above and then incubated in the presence or absence of 1 mM carbachol for 30 minutes prior to indirect immunofluorescence as described in Methods. Images were acquired at 40X.

To examine whether ectopically expressed M_2 _mAChRs undergo agonist-promoted internalization in MEFs, we transiently transfected MEF wild type and corresponding β-arrestin null cells with a plasmid encoding a FLAG-tagged porcine M_2 _mAChR. Following 24 h transfection, MEF wild type, MEF KO1, MEF KO2, and MEF KO1/2 cells were stimulated with 1 mM carbachol for 1 h at 37°C. The number of receptors remaining at the cell surface was measured using a saturating concentration of the hydrophilic ligand [^3^H]-NMS. Approximately 40% of surface M_2 _mAChRs were internalized in wild type MEF cells while M_2 _mAChRs in MEF KO1 and MEF KO2 cells were internalized by 33% and 42%, respectively. In contrast, M_2 _mAChRs were not internalized in MEF KO1/2 (Fig. 2A). These results demonstrated that exogenously expressed M_2 _mAChRs undergo agonist-promoted internalization in MEF wild type cells and either β-arrestin isoform was sufficient for sequestration. To further evaluate where M_2 _mAChRs were localized, we used confocal immunofluorescence microscopy in MEF wild type or MEF KO1/2 cells transiently expressing a FLAG-tagged M_2 _mAChR in the absence or presence of carbachol. As indicated in Figure 2B, diffuse cell surface localization of M_2 _mAChRs was observed prior to carbachol addition in both MEF phenotypes. Upon addition of agonist, M_2 _mAChRs in MEF wild type cells redistributed into discrete intracellular vesicles dispersed throughout the cell while M_2 _mAChRs expressed in MEF KO1/2 cells remained at the cell surface (Fig. 2B). The diffuse pattern shown in MEF KO1/2 cells represents surface plasma membrane localization since the absence of detergent leads to an identical staining pattern as seen in untreated cells (*data not shown*). The FLAG-tag is located at the N-terminus of the receptor and is accessible to exogenously added antibody even in the absence of detergent.

**Figure 3:**
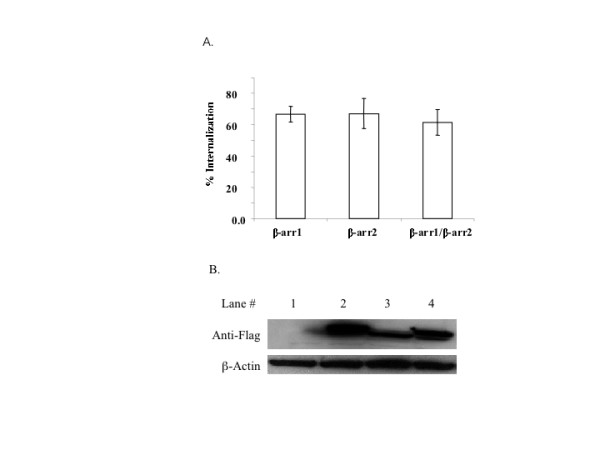
**Expression of β-arrestin 1 or 2 rescued agonist-promoted internalization of M_2 _mAChRs in MEF KO1/2 cells**. Approximately 24 h following co-transfection with constructs encoding M_2 _mAChRs and β-arrestins, cells were stimulated with 1 mM carbachol for 1 h. A.) Agonist-promoted internalization was determined as described in Methods. Data are presented as the mean ± standard deviation of 5 independent experiments consisting of 8–11 determinants. B.) A representative immunoblot of FLAG-tagged β-arrestin and internal protein control β-actin is shown. Lanes consisted of: non-transfected MEF KO1/2 (1), MEF KO1/2 expressing β-arrestin1 (2), MEF KO1/2 expressing β-arrestin 2 (3), and MEF KO1/2 expressing β-arrestin 1 and 2 (4). Western blot shown is a representative of at least 3 independent experiments.

**Figure 4:**
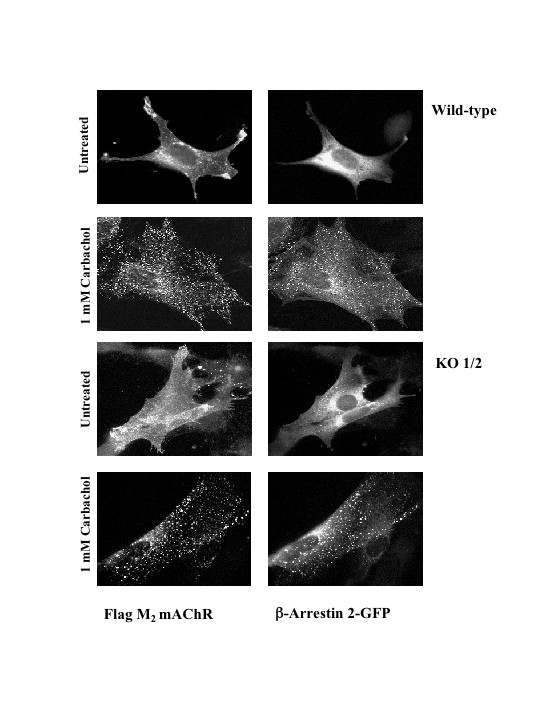
**Stimulation of M_2 _mAChRs leads to stable co-localization of β-arrestin 2-GFP at intracellular sites**. MEF wild type or KO1/2 cells were transiently co-transfected with the human FLAG-tagged M_2 _mAChR and β-arrestin 2-GFP constructs. Following 30 minutes of 1 mM carbachol stimulation, cells were fixed and processed for indirect immunofluorescence as described in the Methods. Localization of β-arrestin 2-GFP and M_2 _mAChR was visualized by confocal microscopy. Confocal images are representative of three independent experiments.

**Figure 5:**
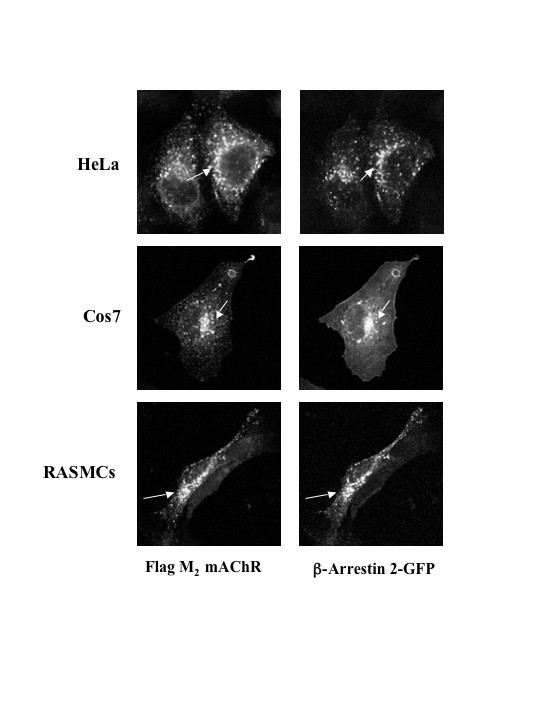
**Agonist-promoted internalized FLAG-tagged M_2 _mAChRs exhibit overlap with β-arrestin 2-GFP at intracellular sites in various cell lines**. Cells were transiently transfected with FLAG-tagged M_2 _mAChR and β-arrestin 2-GFP and treated with 1 mM carbachol for 30 min at 37°C. Co-localization of β-arrestin 2-GFP with internalized M_2 _mAChRs occurred in HeLa, COS-7 and rat aortic smooth muscle cells (RASMCs). Arrows indicate overlap between β-arrestin 2-GFP and M_2 _mAChRs in intracellular compartments. Confocal images are representative of three independent experiments.

To determine whether selectivity existed between β-arrestin isoforms in their ability to mediate agonist-promoted internalization of M_2 _mAChRs, we examined agonist promoted internalization in MEF KO1/2 cells co-expressing M_2 _mAChR and FLAG-tagged β-arrestin 1 and/or 2 (Fig. 3A). Western blotting analysis confirmed that FLAG-tagged β-arrestins were expressed (Fig. 3B). Cells were treated with 1 mM carbachol for 1 h and the extent of receptor internalization was assessed using [^3^H]-NMS. MEF KO1/2 cells reintroduced with β-arrestin 1, β-arrestin 2, or both isoforms exhibited M_2 _mAChR uptake similarly (Fig. 3A). These data suggest that not only is agonist-promoted internalization of M_2 _mAChR β-arrestin-dependent but also there is no selectivity between β-arrestin isoforms (Fig. 2A and 3A). To assess whether stimulated and internalized M_2 _mAChRs co-localize with β-arrestin 1 or 2, we reintroduced GFP-tagged β-arrestin 1, 2, or both isoforms with FLAG-tagged M_2 _mAChRs into MEF KO1/2 cells and assessed their localization by immunofluorescence microscopy. Internalized M_2 _mAChRs remained associated with β-arrestin 1-GFP (*data not shown*) or β-arrestin 2-GFP (Fig. 4) in intracellular compartments following 30 minutes stimulation with 1 mM carbachol. To determine if this phenomenon occurs in other cell types we expressed M_2 _mAChRs in HeLa, COS-7, and rat aortic smooth muscle cells (RASMCs). As observed in MEF KO1/2 cells, internalized M_2 _mAChRs remained co-localized with β-arrestin 2-GFP in HeLa, COS-7, and RASMCs (Fig. 5). These results demonstrate that agonist-promoted internalization of the M_2 _mAChR is β-arrestin-dependent, and that internalized M_2 _mAChRs stably associate with either β-arrestin isoform in multiple cell lines.

**Figure 6:**
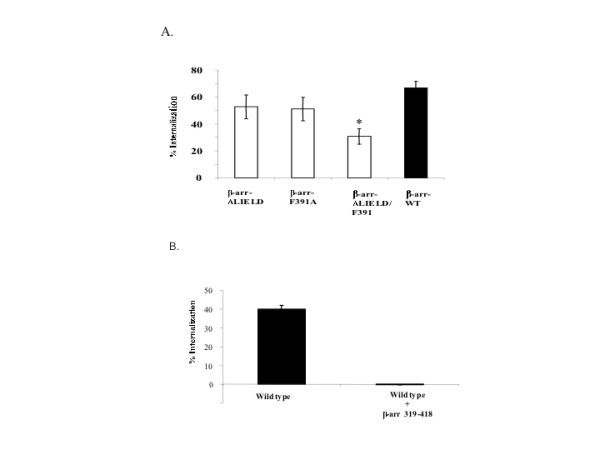
**Expression of β-arrestin mutants deficient in clathrin and/or AP-2 binding interaction partially supports agonist-promoted internalization of M_2 _mAChRs in MEF K/O1/2, while expression of truncated carboxyl-terminal region of β-arrestin 1 (319–418) completely blocked agonist promoted M_2 _mAChR internalization in MEFwt cells**. A.) Approximately 24 hr following co-transfection with FLAG-M_2 _mAChR and β-arrestin 2 clathrin (ΔLIELD), AP-2 (F391A), or clathrin and AP-2 (ΔLIELD/F391A) mutants, MEF KO1/2 cells were stimulated with 1 mM carbachol for 1 h and agonist-promoted internalization was determined as described in Methods. Data are presented as mean ± standard deviation from 4 independent experiments consisting of 8–11 determinants. B.) Approximately 24 hr following transfection with the β-arrestin 1 C-terminal domain (319–418), MEF wild type cells were stimulated with 1 mM carbachol for 1 h and agonist-promoted internalization of receptor was determined as described in Methods. Data are presented as the mean ± standard error from 3 separate experiments with each experiment consisting of 8 to 11 independent determinations. Statistical test was performed using ANOVA with the post hoc Bonferroni/Dunn test (asterisk indicates * p < 0.001).

Previously, sequestration of M_1_, M_3_, and M_4 _mAChRs was shown to be both β-arrestin and clathrin-dependent [23,27]. In contrast M_2 _mAChR sequestration was reported to be largely β-arrestin and clathrin-independent [22,28]. To address whether the β-arrestin-dependent internalization we observed in MEFs was independent of clathrin, we expressed in MEF KO1/2 cells arrestin mutants that were selectively defective in interaction with clathrin (β-arrestin 2 ΔLIELD), AP-2 (β-arrestin 2-F391A), or both clathrin/AP-2 (β-arrestin 2 ΔLIELD/F391A) [29]. Expression of either the β-arrestin 2 ΔLIELD or β-arrestin 2-F391A mutant rescued agonist-promoted M_2 _mAChR internalization (Fig. 6A). However, internalization was only moderately rescued by transient expression of a β-arrestin 2 mutant defective in both clathrin and AP-2 interaction (Fig. 6A). These results indicate that β-arrestin-dependent internalization of M_2 _mAChR may include a component that is independent of interactions between clathrin and AP-2.

Recent studies by Santini and co-workers [30] showed that agonist-mediated activation of the β_2_AR was still capable of inducing recruitment into clathrin coated pits in cells expressing mutant arrestin proteins that were defective in binding with clathrin or AP-2, albeit to a reduced degree. Expression of the truncated COOH-terminal region of β-arrestin 1 (319–418), which contains a clathrin binding site but lacks receptor binding, completely inhibited the β_2_AR mediated clustering of clathrin coated pits [31]. Therefore, we conducted experiments with the truncated β-arrestin 1 (319–418) to determine whether agonist-promoted internalization of the M_2 _mAChR in MEFs would be affected. Transient expression of the truncated β-arrestin 1 (319–418) completely inhibited the agonist-promoted internalization of the M_2 _mAChR in MEF wild type cells (Fig. 6B). Thus, it could be argued that the agonist-promoted internalization of M_2 _mAChR involved a clathrin-dependent pathway. However, as shown previously, expression of an arrestin 2 mutant that was defective in interaction with both clathrin and AP-2 only moderately antagonized the agonist-promoted internalization of M_2 _mAChR in MEF KO1/2 cells (Fig. 6A). It could be argued that this arrestin mutant, defective in clathrin/AP-2 binding, was still capable of interacting with clathrin/AP-2, albeit to a significantly reduced degree. Thus, it is reasonable to conclude that the agonist-promoted internalization of M_2 _mAChRs was clathrin-dependent.

**Figure 7:**
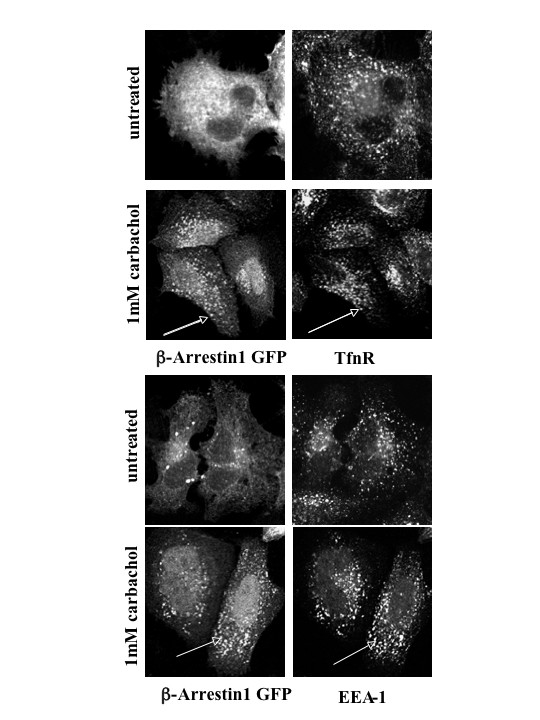
**Addition of agonist leads to the redistribution of β-arrestin 1-GFP to early endosomal structures in the cytosol**. HeLa cells were transiently transfected with human FLAG-tagged M_2 _mAChR and β-arrestin 1-GFP and treated with 1 mM carbachol for 30 minutes. Cells were processed for confocal microscopy. β-arrestin 1-GFP complexes localized to the early endosome as shown by colocalization with markers of that compartment (EEA-1 and TfnR). Arrows indicate signficant overlap between TfnR or EEA-1 with β-arrestin 1-GFP. Confocal images are representative of three independent experiments.

**Figure 8:**
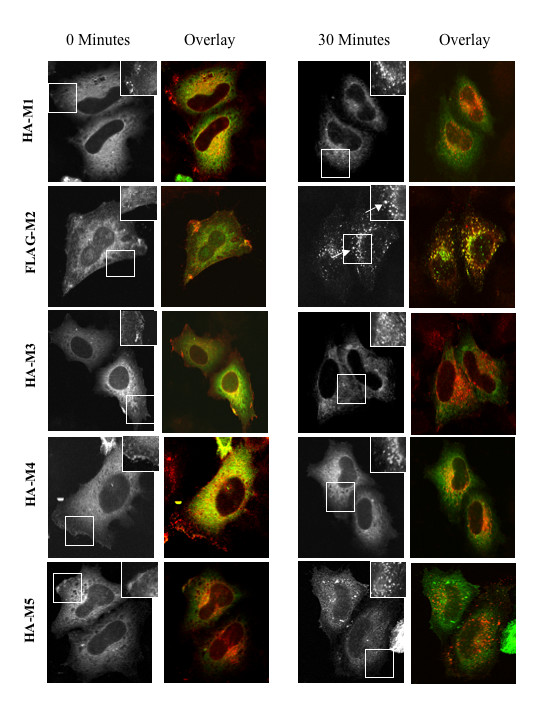
**Internalized M_2 _mAChRs exhibit a differential affinity for β-arrestin 2-GFP compared to other muscarinic subtypes**. HeLa cells were transiently co-transfected with plasmids encoding β-arrestin 2-GFP and either HA-tagged M_1_, M_3, _M_4, _M_5 _mAChR or FLAG-tagged M_2 _mAChR. Cells were untreated or treated with 1 mM carbachol for 0 min or 30 min. Grayscale image indicates β-arrestin 2-GFP localization while the upper right inset indicates immunostaining of the mAChR in a small section of the cell (outline). Arrows indicate overlap between internalized M_2 _mAChRs and β-arrestin 2-GFP. Overlay represents co-immunostaining of mAChR (red) and β-arrestin 2-GFP expression (green) and their colocalization (yellow).

Based upon the findings described above, we sought the identity of the endosomal structures to which β-arrestin localized following M_2 _mAChR activation. We performed co-localization analyses using markers of the early endosome, the early endosomal autoantigen-1 (EEA-1) and the transferrin receptor (TfnR), in combination with β-arrestin 1-GFP. β-arrestin 1-GFP and FLAG-M_2 _mAChRs were co-expressed in HeLa cells, and cells were stimulated with 1 mM carbachol for 30 minutes. Our results showed that β-arrestin 1-GFP significantly co-localized with EEA-1 and TfnR (as indicated by arrows in Fig. 7). β-arrestin 1-GFP was not observed to be associated with EEA-1 or TfnR in unstimulated HeLa cells (Fig. 7). These results indicate that once M_2 _mAChRs are internalized via a β-arrestin dependent pathway, they remain co-localized with β-arrestin in clathrin-derived early endosomes. To address whether other muscarinic receptor subtypes stably associate with β-arrestin in endosomes, we co-expressed HA-tagged M_1, _M_3, _M_4, _and M_5 _mAChRs with β-arrestin 2-GFP in MEF wild type cells and assessed β-arrestin localization using confocal microscopy (Fig. 8). The upper right inset in each frame of the figure shows the localization of the muscarinic receptor subtype for a small section of the cell co-expressing β-arrestin 2-GFP. Overlay images indicate co-immunostaining of mAChRs (red) with β-arrestin 2-GFP (green) and their extent of co-localization (yellow). In the absence of carbachol, β-arrestin 2-GFP was diffusely localized in the cytosol of cells expressing M_1 _– M_5 _mAChR subtypes (Fig. 8, 0 min). Following 30 minute carbachol stimulation only cells expressing human FLAG-tagged M_2 _mAChRs exhibited β-arrestin 2-GFP localization in intracellular compartments as shown by arrows indicating overlap and corresponding overlay image (Fig. 8, 30 min); in cells expressing other receptor subtypes, β-arrestin 2-GFP remained diffusely distributed. Hence, only cells expressing the FLAG-tagged M_2 _mAChR subtype exhibited a stable interaction with β-arrestin at intracellular sites compared to the other muscarinic subtypes.

## Discussion

In the present study, we investigated the role of β-arrestin in agonist-promoted internalization of the M_2 _mAChR, which has previously been reported to be β-arrestin independent. In previous studies, heterologous over-expression of wild type and dominant-negative forms of arrestins was used to assess the function of these proteins [22,32]. Unfortunately, such studies are difficult to interpret because of the complications associated with endogenous proteins. In an attempt to alleviate these complications, we utilized mouse embryonic fibroblasts (MEFs) derived from β-arrestin knockouts in which endogenously expressed β-arrestin 1 and 2 have been genetically eliminated [26]. These cells provide us a unique opportunity to assess whether β-arrestin proteins are involved in the process of agonist-promoted internalization of M_2 _mAChRs. Herein, we show that agonist-promoted endocytosis of the M_2 _mAChR is β-arrestin- and clathrin-dependent.

Both β-arrestin 1 and 2 isoforms were reported to form high affinity complexes with the agonist-activated M_2 _mAChR [33], suggesting that either isoform is capable of mediating agonist-promoted internalization of the receptor. In corroboration with these findings, we observed no selectivity between β-arrestin isoforms in mediating agonist-promoted internalization of M_2 _mAChRs. Perhaps, this lack of selectivity between β-arrestin 1 and 2 may explain why using over-expression of a single mutant form of β-arrestin fails to completely block the agonist-promoted internalization of M_2 _mAChRs. Interestingly, our studies further revealed that β-arrestin remained stably associated with the M_2 _mAChR in juxtanuclear endosomes for prolonged periods of time following agonist exposure. Given that MEF cells do not endogenously express mAChRs, we compared our observations in a physiologically relevant cell line (RASMCs) and two model cell lines (HeLa and COS-7). Similar findings were also observed in these cells. Closer examination of β-arrestin post-endocytic trafficking revealed that M_2 _mAChR stimulation led to arrestin redistribution into Tfn and EEA-1 positive compartments, markers of the early endosome. In accordance with our findings, Delaney *et al*. have reported that stimulated M_2 _mAChRs internalize in a manner that quickly merges with clathrin-derived early endosomes [25].

M_2 _mAChRs follow the general pattern utilized by most GPCRs in that they are internalized via a β-arrestin-dependent mechanism. Additionally, the stable binding of β-arrestin with activated M_2 _mAChRs within microcompartments follows the paradigm of other Class B GPCRs. Implications of these findings are that β-arrestin may dictate the intracellular trafficking and/or signalling of the M_2 _mAchRs. Since β-arrestin has emerged as a versatile adaptor and scaffolding protein, its role in regulating M_2 _mAChR-dependent cellular activity may be significant. It has been shown that β-arrestins interact with trafficking machinery such as Arf6, RhoA, NSF, and a variety of signalling proteins such as ASK1, JNK3, and ERK1/2 [34]. Stable β-arrestin/receptor complexes, as exhibited by Class B receptors, appear to redirect signalling complexes to the cytoplasm thereby activating cytoplasmic targets while preventing ERK translocation to the nucleus [15,16,35]. The physiological role of this process may be to participate in actin cytoskeleton reorganization and chemotaxis [18,36]. With regard to intracellular trafficking, patterns of β-arrestin binding to activate receptors appear to modulate receptor recycling and/or degradation [37]. Class A receptors are typically resensitized and subsequently recycled while Class B receptors undergo slow recycling and/or down-regulation. M_2 _mAChRs have been shown to undergo slow recycling back to the plasma membrane upon agonist removal [38]. What role or roles β-arrestin plays in M_2 _mAChR recycling and/or degradation is currently unknown. The functional consequence of stable β-arrestin/M_2 _mAChR complexes remains to be determined.

Previous studies have suggested that M_2 _mAChR internalization does not proceed through a β-arrestin/clathrin mediated pathway [22,23,28]. For example, Delaney and co-workers [25] previously reported that M_2 _mAChRs internalized by a clathrin-independent pathway based upon the use of a dominant-negative K44A dynamin-1 mutant. However, expression of a N-terminal deletion dynamin-1 mutant N272 that lacks the complete GTP-binding domain, unlike K44A dynamin, strongly inhibited agonist-promoted M_2 _mAChR internalization [39]. Therefore, we conducted experiments with arrestin mutants that were selectively deficient in interaction with clathrin, AP-2, or both clathrin and AP-2, to determine whether agonist mediated internalization of M_2 _mAChRs was clathrin-dependent. Expression of arrestin mutants defective in interaction with either clathrin (β-arrestin 2-ΔLIELD) or AP-2 (β-arrestin 2-F391A) failed to antagonize M_2 _mAChR internalization. Moreover, over-expression of a dominant-negative arrestin mutant that was defective in interaction with both clathrin and AP-2 only modestly antagonized M_2 _mAChR internalization in MEF KO1/2 cells. Thus, it is reasonable to conclude that these data corroborate previous studies indicating that M_2 _mAChR internalization is clathrin-independent. However, Santini and co-workers [30] have reported that arrestin mutants with impaired binding to clathrin or AP-2 were still capable of displaying recruitment of β_2_AR to clathrin-coated pits, albeit to a reduced degree. Therefore, it may be premature to conclude that M_2 _mAChR internalization is β-arrestin-dependent but clathrin/AP-2-independent. Expression of the truncated carboxy-terminal region of β-arrestin 1, which contained the clathrin interaction site, has been shown to completely abrogate β_2_AR mediated clustering of clathrin coated pits [31]. Exogenous expression of this mutant completely block agonist-promoted internalization of M_2 _mAChRs in wild type MEFs. Collectively, these results indicate that agonist-promoted internalization of M_2 _mAChRs is β-arrestin-dependent and most likely clathrin/AP-2-dependent. However, we cannot rule out that the C-terminal region of arrestin 1 is interacting with another factor, independent from clathrin/AP-2 that may be responsible for mediating internalization of the M_2 _mAChR. Indeed previous studies have shown that the Arf6 GTPase regulates agonist-promoted endocytosis of the M_2 _mAChR [24,25]. It has been shown that β_2_AR stimulation leads to activation of Arf6 GTPase, which facilitates receptor endocytosis [40]. It is feasible that sequestration of M_2 _mAChR requires activation of Arf6 GTPase by a β-arrestin-mediated pathway, which may be an important component of agonist-promoted internalization of the M_2 _mAChR. This would corroborate previous studies, which indicate a critical role for Arf6 GTPase in mediating agonist-promoted M_2 _mAChR internalization [24].

The differential trafficking of β-arrestin with mAChRs to endosomes appears to be subtype specific. There are five muscarinic subtypes termed M_1_mAChR- M_5 _mAChR. M_1_, M_3, _and M_5 _mAChRs couple to G_q _proteins and activate phospholipase C whereas M_2 _mAChR and M_4 _mAChR couple to G_i/o _to inhibit adenylyl cyclase and activate K^+ ^channels [1,2]. As shown in Figure 8, stimulated muscarinic subtypes aside from M_2 _mAChRs are sequestered into endocytic vesicles that are devoid of β-arrestin. It has been shown that M_1 _mAChR, M_3 _mAChR, and M_4 _mAChR require β-arrestin in mediating agonist-promoted internalization [23] so we do not rule out the possibility that arrestin is recruited to the plasma membrane following stimulation and then rapidly disassociates from the receptor. It is possible that carbachol may induce receptor conformations that may not promote stable β-arrestin associations with the other mAChR subtypes. However, sequence alignment of the M_2 _and M_4 _mAChR (using the T-coffee program) revealed that the subtypes exhibit high sequence similarities; interestingly, the sequence differences lie in the third intracellular loop, specifically at residues 293–313 within the M_2 _mAChR. As described by Pals-Rylaarsdam and others, a cluster of serine and threonine sites at positions 307–311 undergo agonist promoted phosphorylation, which is necessary and sufficient for β-arrestin interaction [6]. This site may be important for designating stable interactions with β-arrestin. M_2 _mAChR sequences downstream from this site at 348–368 also differ significantly from the M_4 _mAChR suggesting that an additional motif may be involved.

## Conclusion

In summary, the data presented in this study demonstrate that the agonist-promoted endocytosis of the M_2 _mAChR subtype occurs via an arrestin dependent pathway in MEF cells. Exogenously expressed β-arrestin proteins remained stably associated with the M_2 _mAChR upon entry into early endosomal compartments. The lack of stable β-arrestin interaction with other mAChR subtypes suggests a unique role of β-arrestin in regulating activity of the M_2 _mAChR subtype.

## Methods

### Materials

[^3^H]-*N*-methylscopolamine (NMS) (81–84 Ci/mmol) was purchased from Amersham Corp. (Buckinghamshire, England). Dulbecco's Modified Eagle's Medium (DMEM), F-10, penicillin/streptomycin, fetal bovine serum, restriction enzymes and LipofectAMINE 2000 were purchased from Invitrogen (Carlsbad, CA) EX-GEN was purchased from Fermentas (Hanover, MD). The anti-FLAG M2 monoclonal antibody and mouse anti-M1 FLAG antibody were purchased from Sigma-Aldrich (St. Louis, MO); mouse antibodies against β-arrestin 1 and 2 were purchased from Santa Cruz (Santa Cruz, CA). The anti-HA.11 monoclonal antibody was purchased from Covance Research Product (Berkley, California) Secondary HRP-conjugated antibodies were purchased from Jackson Immunoresearch Laboratories Inc. (West Grove, PA). Carbachol, atropine and all other reagents were purchased from Sigma-Aldrich. Dr. Neil Nathanson (University of Washington) kindly provided the construct expressing the porcine FLAG-tagged M_2 _mAChR [32]. HA-tagged M_1_, M_3_, M_4_, and M_5 _mAChRs were purchased from UMR cDNA Resource Center (University of Missouri). Arrestin mutants, β-arrestin 2-ΔLIELD, β-arrestin 2-F391A, β-arrestin 2 ΔLIELD/F391A, and truncated carboxyl-terminal region of β-arrestin 1 (319–418) were kindly provided by Dr. Jeffrey Benovic (Thomas Jefferson University) [30,31]. The MEF wild type, β-arrestin 1 and 2 single knockouts, β-arrestin 1 and 2 double knockout cells, and constructs for FLAG-tagged β-arrestin 1 and 2 were kindly provided by Dr. Robert Lefkowitz (Duke University Medical Center) [26]. Constructs encoding β-arrestin 2-GFP and β-arrestin 1-GFP were generous gifts from Dr. Stefano Marullo and have been previously described [41].

### Cell Culture and Transient Transfection

HeLa, MEF wild-type, MEF single and double β-arrestin knockout, RASMCs, and COS-7 cells were maintained in DMEM supplemented with 10% fetal bovine serum (FBS), 100 I.U./ml penicillin, and 100 μg/ml streptomycin at 37°C with 5% CO_2_. For immunocytochemistry, HeLa cells were grown on glass coverslips at a density of 120,000 cells/well in six-well dishes and transfected with EX-GEN or LipofectAMINE 2000 according to the manufacturer's protocol using 1 μg of DNA/well. For ligand binding assays, MEF cells were plated at 80,000 cells/well in 24 well plates and transfected with EX-GEN or LipofectAMINE 2000 according to the manufacturer's protocol using 1 μg of DNA/well.

### Radioligand Binding Assay

Receptor internalization was determined by measuring the binding of the membrane impermeable muscarinic antagonist [^3^H]-*N*-methylscopolamine ([^3^H]-NMS) to intact cells as previously described [42]. Briefly, 24–42 h after transfection, MEF cells cultured in 24-well plates were treated or not treated with 1 mM carbachol for 60 min at 37°C. Cultures were washed twice with 1 ml of ice-cold PBS, and labelled with 720 fmol of [^3^H]-NMS in 1 ml PBS for 4 h at 4°C. Non-specific binding was determined as the bound radioactivity in the presence of 1 μM atropine. Labelled cells were washed two times with 1 ml of ice-cold PBS, solubilized in 0.5 ml of 1% Triton X-100 and combined with 3.5 ml of scintillation fluid followed by measurement of radioactivity. Receptor internalization is defined as percent of surface M_2 _mAChRs not accessible to [^3^H]-NMS at each time relative to non-carbachol-treated cells.

### Immunoblotting

Western blot analysis was performed on cells cultured in 6-well plates. The cells were solubilized in 0.5 ml of lysis buffer containing: 50 mM HEPES (pH 7.5), 0.5% (v/v) Nonidet P-40, 250 mM NaCl, 2 mM EDTA, 10% (v/v) glycerol, 1 mM sodium orthovanadate, 1 mM sodium fluoride and 1 μg/ml of protease inhibitors leupeptin, aprotinin, pepstatin A, and 100 μM benzamidine. The protein concentration was determined using the Bradford assay method. Fifty μg of cell lysates were subjected to 4–20% SDS-PAGE. After transfer, the nitrocellulose membrane was blocked and then probed with anti-FLAG monoclonal antibody. Immunoreactive bands were visualized by enhanced chemiluminescence after adding HRP-conjugated anti-mouse antibody. After stripping with 0.1 M glycine (pH 2.5), the membrane was re-probed with anti-β-actin using a detection kit from Oncogene (Cambridge, MA).

### Indirect Immunofluorescence

24 h following transfection, cells were treated as described in the figure legends, fixed in 4% formaldehyde in PBS for 5 minutes, and rinsed with 10% fetal bovine serum and 0.02% azide in PBS (PBS/serum). Fixed cells were incubated with primary antibodies diluted in PBS/serum containing 0.2% saponin for 45 minutes, and then washed with PBS/serum (3 × 5 min.). The cells were then incubated with fluorescently labelled secondary antibodies in PBS-serum and 0.2% saponin for 45 minutes, washed with PBS/serum (3 × 5 min.) and once with PBS, and mounted on glass slides. Images were acquired using a Zeiss LSM 510 scanning confocal microscope or an Olympus BX40 epifluorescence microscope equipped with a 60× Plan pro lens, and photomicrographs were prepared using an Olympus MagnaFire SP digital camera (Olympus America, Inc.). All images were processed with Adobe Photoshop 7.0 software.

### RNA Isolation and RT-PCR

Total cellular RNA from MEF cells, cortex and cerebellum of 2–3 week old Sprague Dawley rat pups was isolated using TriZol according to the manufacturer's instructions. A 50 μl reaction solution containing 1 μg total RNA was reverse-transcribed, and PCR was performed using gene-specific primers and the Qiagen One-step RT-PCR kit. Gene specific primers and amplification reactions were as follows: Rat M_1 _mAChR (175 bp amplified product): CCTCTGCTGCCGCTGTTG (sense) and GGTGGGTGCCTGTGCTTCA (antisense); Rat M_2 _mAChR (686 bp amplified product): CACGAAACCTC TGA CCTACCC (sense) and TCTGACCCGACGACCCAACTA (antisense); Rat M_4 _mAChR (587 bp amplified product): TGGGTCTTGTCCTTTGT GCTC (sense) and TTCATTGCCTGTCTGCTT TGTTA (antisense); Rat β-actin (764 bp amplified product): TTGTAACCAACTGGGACGATATGG (sense) and GATCTT GATCT TCATGGT GCTAGG (antisense). Cycling parameters were 30 minutes at 50°C for reverse transcription followed by 1 minute 95°C hot start followed by 28 cycles at 95°C for 1 minute, 62°C for 1 minute, and 72°C for 45 seconds and a final cycle at 72°C for 7 minutes.

## Competing interests

The author(s) declare that they have no competing interests.

## Authors' contributions

KTJ and ME carried out the radioligand binding, western blot analysis, immunocytochemistry, and draft of the manuscript. AG carried out the RT-PCR experiments. VM helped to draft the manuscript. DAJ conceived of the study, participated in its design and coordination, performed the statistical analysis and helped to draft the manuscript. All authors read and approved the final manuscript.
